# Screening and evaluation of skin potential probiotic from high-altitude Tibetans to repair ultraviolet radiation damage

**DOI:** 10.3389/fmicb.2023.1273902

**Published:** 2023-10-19

**Authors:** Zhihao Zhang, Haixia Ran, Yutong Hua, Feilong Deng, Bo Zeng, Jianmin Chai, Ying Li

**Affiliations:** ^1^Guangdong Provincial Key Laboratory of Animal Molecular Design and Precise Breeding, College of Life Science and Engineering, Foshan University, Foshan, China; ^2^School of Life Science and Engineering, Foshan University, Foshan, China; ^3^Animal Husbandry and Fisheries Technology Extension Station, Chongqing, China; ^4^Farm Animal Genetic Resources Exploration and Innovation Key Laboratory of Sichuan Province, Sichuan Agricultural University, Chengdu, China

**Keywords:** skin microbiota, skin disease, UV damage, high altitude, culturomics, probiotic

## Abstract

Human skin microbes play critical roles in skin health and diseases. Microbes colonizing on the skin of Tibetans living in the high-altitude area for generations may have a stronger ability to resist the harsh environment, such as high ultraviolet radiation (UV). Isolation of a potential probiotic from Tibetans skin is beneficial for resistance of skin disease for humans in the world. In this study, the signature microbiota for Tibetan skin were characterized compared to low-altitude humans. Next, using culture-omics, 118 species were isolated. The culturability of high-altitude of Tibetan skin microbiome reached approximate 66.8%. Next, we found that one strain, *Pantoea eucrina*, had the greatest ability to repair UV damage to the skin as the lowest pathological score was observed in this group. Interestingly, another animal trial found this bacterium resisted UV rather than its metabolites. Using whole genome sequencing, this strain *P. eucrina KBFS172* was confirmed, and its functions were annotated. It might involve in the metabolic pathway of carotenoid biosynthesis with anti-oxidative stress properties, which plays critical roles in UV-damage repair. In conclusion, we characterized the signature microbes of skin in high-altitude Tibetans, isolated a skin bacterium of *Pantoea eucrina KBFS172* which could repair UV damage via involving the metabolic pathway of carotenoid biosynthesis. Our results provide a new potential skin probiotic for skin disease prevention or sunburn.

## Introduction

Skin, the largest organ of the human body, has over 30 m^2^ surface area, and serves as the first line to defense against the harsh external environment (Yoshio et al., 2004; Ross et al., 2017). Variable microbes colonize on the surface of skin (Gallo, 2017). Advances in sequencing and bioinformatics have unraveled the mysteries of the skin microbiome (Gao et al., 2008; Grice et al., 2008), including the characterization of commensal and pathogenic microorganisms on human skin (Roszkowski et al., 1990; Tang and Stratton, 2010). Numerous studies have demonstrated the crucial roles of skin commensal bacteria in maintaining the overall health of the skin and the host (Shu et al., 2013; Wang et al., 2014; Knackstedt et al., 2020). However, the specific roles of skin microbiota associated with common diseases (e.g., skin cancer) are still unclear.

Qinghai-Tibet Plateau (QTP), known as “the roof of the world,” is the highest plateau on earth, with a resident population exceeding 12 million as early as 2006 (Li et al., 2019). The living environment in QTP is extreme, with high ultraviolet radiation (UV), hypobaric pressure, and hypoxia (Jablonski and Chaplin, 2010). In spite prolonged exposure to high-intensity UV may cause skin cancer (Armstrong et al., 2001), the adaptation of Tibetans may have ability to resist extreme environment (e.g., UV) or even skin cancer. In term of this, some studies started to investigate the skin microbiota in subjects living in high and low altitudes. Previous studies have shown that high altitudes impacted the composition and structure skin microbes (Muletz Wolz et al., 2018; Li et al., 2019). Our previous study suggested that altitudes have a significant effect on the skin microbiome of Tibetans (Zeng et al., 2017). However, whether commensal bacteria on the skin of Tibetans could resist UV and what is the mechanism are still unknown.

Thus, we hypothesized that skin microbiota isolated from humans living in QTP may have the ability to resist and repair the UV damage. In this study, we first compared the human skin microbiomes form high and low altitude by re-analyzing our previous published data. Then we isolated and purified high-altitude Tibetan skin microbes using the culturomics method, and validated the potential functions of bacterial isolations on the repair of the UV damage by a mice model. Furthermore, we utilized whole-genome sequencing to unveil the mechanism of this isolate to resist UV. This current isolated a potential skin probiotic successfully, which benefits the protection of disease, such as skin cancer and sunburn.

## Materials and methods

The experiment protocol was approved by the Animal Ethics and Humane Animal Care of the Foshan University (protocol#: FOSU2021010).

### Sample collection

Ten healthy Tibetans (Supplementary Table S1), living in the Daocheng area (high altitude, 3,750 masl, latitude 27°58’ N, longitude 99°56′ E) in China and having long-term outdoor activities (e.g., agricultural production), were recruited for this study. All human subjects had no skin disease and free access of antibiotics within 3 months when sampled. All the participants were given the written informed consent. Relevant characteristics, including gender, age, and sampling site, were recorded to exclude individuals with interfering factors in this experiment. Skin samples were collected by swabbing the forehead of the Tibetans. Regarding sampling, all sterile swabs were pre-wet with SCF-1 buffer [50 mM Tris buffer [pH 7.6], 1 mM EDTA [pH 8.0], 0.5% Tween-20], and were rubbed vigorously on the forehead over 30 times to collect skin microbes. Then, the forehead swabs were cut off and placed into 2 mL centrifuge tubes, snap-frozen in liquid nitrogen, and stored at −80°C until further analysis.

### Pure culture

The samples were thawed from −80°C and transferred to 4°C for 30 min. After the microorganisms were revived, the swab heads were immersed in sterile PBS buffer in the EP tube to shake out the bacteria. The resulting bacterial suspension was used for further pure culture experiments.

Five culture media, namely Nutrient Agar (Van der Weele et al., 2000), R-2A Agar (Reasoner et al., 1985; Massa et al., 1998), TSBYS (Peeters et al., 2011; Nicholson et al., 2013), BHIA (Osawa, 1990) and Agar medium J (Pagnanelli et al., 2000), were selected for pure culture. Each medium had six replicates. The bacterial suspension was mixed well with sterile PBS buffer (pH = 7.0) at a ratio of 0.1:9.9. Then, 200 μL of the bacterial suspension was spread evenly onto each prepared culture medium plate using a coating rod. The plates were then incubated in an inverted position at 28°C for microbial growth. The plates were observed every 12 h until no new colonies appeared. Three plates of each culture medium for each sample were randomly selected for subculture, and the plates were eluted with sterile PBS buffer. The eluted liquid was then sequenced directly to obtain the V3-V4 region of the 16S rRNA gene.

### DNA extraction, sequencing, and bioinformatics for pure culture sample

For pure culture samples, the DNA of all the single strain were extracted using the boiling method (Dashti et al., 2009). The full-length sequence of 16S rRNA gene was amplified using the 27f/1492r primers (27f: 5’-AGAGTTTGATCCTGGCTCAG-3′, 1492r: 5’-TACGGYACCTTGTTACGACTT-3′). The quality of the PCR products was assessed by 1% gel electrophoresis, and qualified samples were sequenced on an Illumina MiSeq platform (Sangon Biotech Co., Ltd., Shanghai, China). Raw sequencing reads were quality controlled using Seqman software (Swindell and Plasterer, 1997). The high-quality sequences were aligned using Blastn in the NCBI database, and similar sequences of genes of single bacteria and their similarities were obtained. The isolated strains were then preliminarily divided into groups.

For the non-culture group that was sequenced directly from the swab heads, the total bacterial DNA was extracted using a Mo Bio PowerFecal DNA isolation kit according to the manufacturer’s recommendations. DNA concentration (μg/μl) was quantified using a NanoDrop 2000C ultra-micro spectrophotometer. The general primers 341F (5’-CCTAYGGGRBGCASCAG-3′) and 806R (5’-GGACTACNNGGGTATCTAAT-3′) were used to amplify the V3-V4 region of the 16S RNA gene. The quality of the PCR products was checked by 1% gel electrophoresis, and qualified samples were sequenced on an Illumina MiSeq platform (NovogeneCo, Ltd., Beijing, China). The raw sequences were denoised, dereplicated, and filtered for chimeras using QIIME2 (Bolyen et al., 2019) (dada2 Package). The previous data (Zeng et al., 2017) (16S rRNA, v4 region, 128 samples) were also quality controlled using QIIME2 (deblur Package) (Bolyen et al., 2019). The taxonomy was annotated using the Greengenes database (gg_13_8) (Cannone et al., 2002).

### UV irradiation trial #1

Five-week-old SPF-class female ICR mice (*n* = 38, body weight = 23 g) were used for this trial. The mice were divided into eight groups: a blank control group (*n* = 3) that received no treatment, negative control group (*n* = 5), and experimental groups A–E (*n* = 5 for each group) that received bacterial therapy with different candidates (A: *Arthrobacter gandavensis*; B: *Bacillus psychrosaccharolyticus*; C: *Pantoea eucrina*; D: *Paenibacillus amylolyticus*; E: *Paenibacillus terrae*) (Supplementary Table S3). After a preparation period, the area on the back of each mouse, measuring 2 cm × 3 cm, was depilated and smeared with the corresponding bacterial suspension for 3 days.

Before the trial, the mice were pre-feed with SPF pellet feed for one week. All feeding utensils were sterilized twice a week, and the ambient temperature was controlled at 20–25°C. Each mouse was housed in an individual cage with *ad libitum* access to food and water. After the mice were adaptively fed for a week, and bacterial colonization of each mouse was checked by bacterial culture of the back. Further details regarding the grouping and treatment conditions can be found in Supplementary Table S3. All mice were exposed to the same combination-rays light, and the changes in the skin morphology of each group were observed and recorded daily throughout the trial. Two types of UV light (UVA, UVB) boxes were used to irradiate the depilated mice.

A UV facility comprises a UVA lamp (40 W, with a wavelength range of 320 to 400 nm and a peak wavelength of 365 nm, from Philips, Germany) and a UVB lamp (20 W, with a wavelength range of 290 to 320 nm and a peak wavelength of 297 nm, also from Philips, Germany) positioned side by side. The distance between the lamps and the backs of the mice was set at 30 to 40 cm, and the irradiation intensity was measured using a UV-radiometer (UVAB/ST-513, SENTRY, Taiwan, China). We followed the subacute light injury model used in animal experiments (Wei et al., 2002) and optimized it based on our specific experimental conditions. After preheating the tubes for 20 min, the mouse cages were placed under the lamp box. During the trial, the position of the mouse cages was rotated to ensure equal irradiation dose for all mice. In the first week of irradiation, we performed daily UV irradiation, with a daily UVA dose of 3.96 J/cm^2^ and a UVB dose of 252 mJ/cm^2^. During the second and third weeks of irradiation, we performed UV irradiation every other day for a duration of 6 h, with UVA and UVB doses of 10.8 J/cm^2^ and 1.08 J/cm^2^, respectively. After each irradiation, we applied the corresponding bacterial suspension on the depilated area of the mice’s backs. Overall, the total amount of UVA irradiation in this trial was 114.12 J/cm^2^, and for UVB, it was 8.964 J/cm^2^.

**Figure 1:**
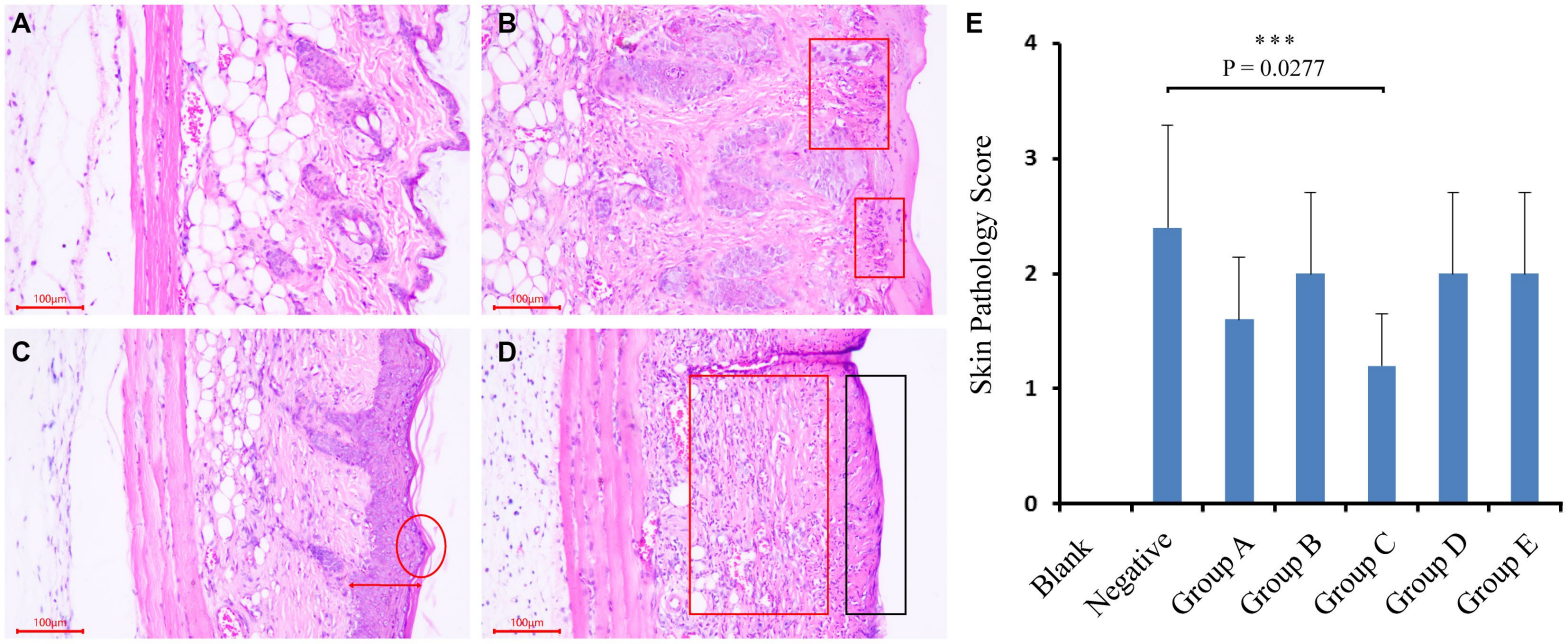
Mice trial #1: pathological changes of skin and sections, and scoring of skin pathological changes. **(A–D)** As followed, no obvious pathological changes in mouse skin; mild lesions, a small amount of inflammatory cell infiltration; moderate lesions, epidermal thickening, inflammatory cell infiltration and severe lesions, exposed subcutaneous tissue, and a large number of inflammatory cell infiltration. **(E)** Scores of pathological changes in skin sections of mice in each group. Blank (blank control group): without UV and smear bacteria treatment; Negative (negative control group): only UV treatment without smear bacteria suspension treatment; Group A–E: separately smear the suspension of *Arthrobacter gandavensis, Bacillus psychrosaccharolyticus, Pantoea eucrina, Paenibacillus amylolyticus*, and *Paenibacillus terrae.*

At the end of the trial, the mice were euthanized by cervical dislocation. The skin on their backs was immediately peeled off and immersed in a 4% formaldehyde solution, and stored at 4°C. The skin was then cut into 5 μm thickness sections, stained with H&E (hematoxylin–eosin staining), and observed under a microscope to record the histopathological changes (Figures 1A–D).

### Mice UV irradiation trial #2

*Pantoea eucrina* has been shown to significantly repaired the UV damage by the first trial, we thus designed the second validation trial. Five-week-old SPF-class female ICR mice (*n* = 40) were used for this trial. The mice were divided into five groups, with eight mice per group: BS group (bacterial suspension: Suspension of *Pantoea eucrina*), BL group (bacterial lysate: lysate of *Pantoea eucrina*), NC group (negative control), and VE group (vitamin E). To prepare the bacterial suspension, the bacteria were activated and then incubated overnight in nutrient broth with shaking at 28°C. The concentration of the suspension was adjusted to 1 × 10^8^ CFU/mL by counting on the plate. To prepare the bacterial lysate, the bacterial suspension was taken into a centrifuge tube and subjected to three cycles of freeze-thawing (−20°C, 30 min; 20°C, 20 min). The bacteria were then lysed using an ultrasonic cell disruptor (JY96-IIN, ShangHai JinXin). The lysate was then cooled in an ice-water bath for 30 min, filtered through a 0.22 μm filter, and stored at 4°C. For further details on the experimental grouping and treatment conditions, please refer to Supplementary Table S4. The trial procedures were the same as those of the previous trial (Mice UV Irradiation Trial #1).

After the trial, the skin on the back of the mice was stained using the same method as in the previous trial. The observations and recordings were conducted in a similar manner.

### The whole genome sequencing

The bacterial specie (*Pantoea eucrina*) in group C were isolated to perform whole genome sequencing in order to identify the bacterial strain and its potential functions. The sequencing was performed on a single strain using the Nanopore sequencing technology platform. The raw data was subjected to format conversion and filtering. Canu v1.5 (Koren et al., 2017) was utilized for assembling the filtered subreads and Pilon (Walker et al., 2014) was used to correct any errors in the assembled genomes. Prodigal (Hyatt et al., 2010) was employed to predict the encoded genes in the assembled genome. The default parameters for these three softwares were chosen. Finally, the assembled contig sequences were aligned with the NT database to determine the chromosome type.

The predicted gene sequences were aligned using the BLAST algorithm (Altschul et al., 1997) to the Non-Redundant Protein Database (Nr database). Subsequently, the functional annotation of the Gene Ontology (GO) database (Ashburner et al., 2000) was performed using the Blast2GO software (Conesa et al., 2005) based on the alignment results with the Nr database. In addition, KEGG (Kanehisa et al., 2004) metabolic pathway enrichment analysis and GO functional enrichment analysis were carried.

### Macroscopic and microcosmic evaluation

The changes in the mice’s skin were observed and recorded daily, and the degree of skin damage on their backs was evaluated based on Supplementary Table S5. After the UV Irradiation Trial, the mice were sacrificed, and pathological sections of their backs were made. The degree of pathological changes was evaluated using Supplementary Table S6 as a reference.

### Statistical analyses

The algorithm of Linear Discriminant Analysis (LDA) coupled with effect size (LEfSe) (Paulson et al., 2013) was employed to determine features with significantly different abundances between high and low altitude human groups by re-analyzing our previous data (SRP065099, Sequence Read Archive (SRA) in NCBI).

The alpha and beta diversity of the high-altitude Tibetan skin microbiome of the 16S sequencing data were measured using the QIIME2 platform. The differences in alpha and beta diversity between groups were calculated using Student’s t-test. Statistical significance was determined at *p* < 0.05 for all analyses. All figures were generated using the R (Wickham, 2011).

## Results

### Skin microbial differences in high- and low-altitude humans

**Figure 2:**
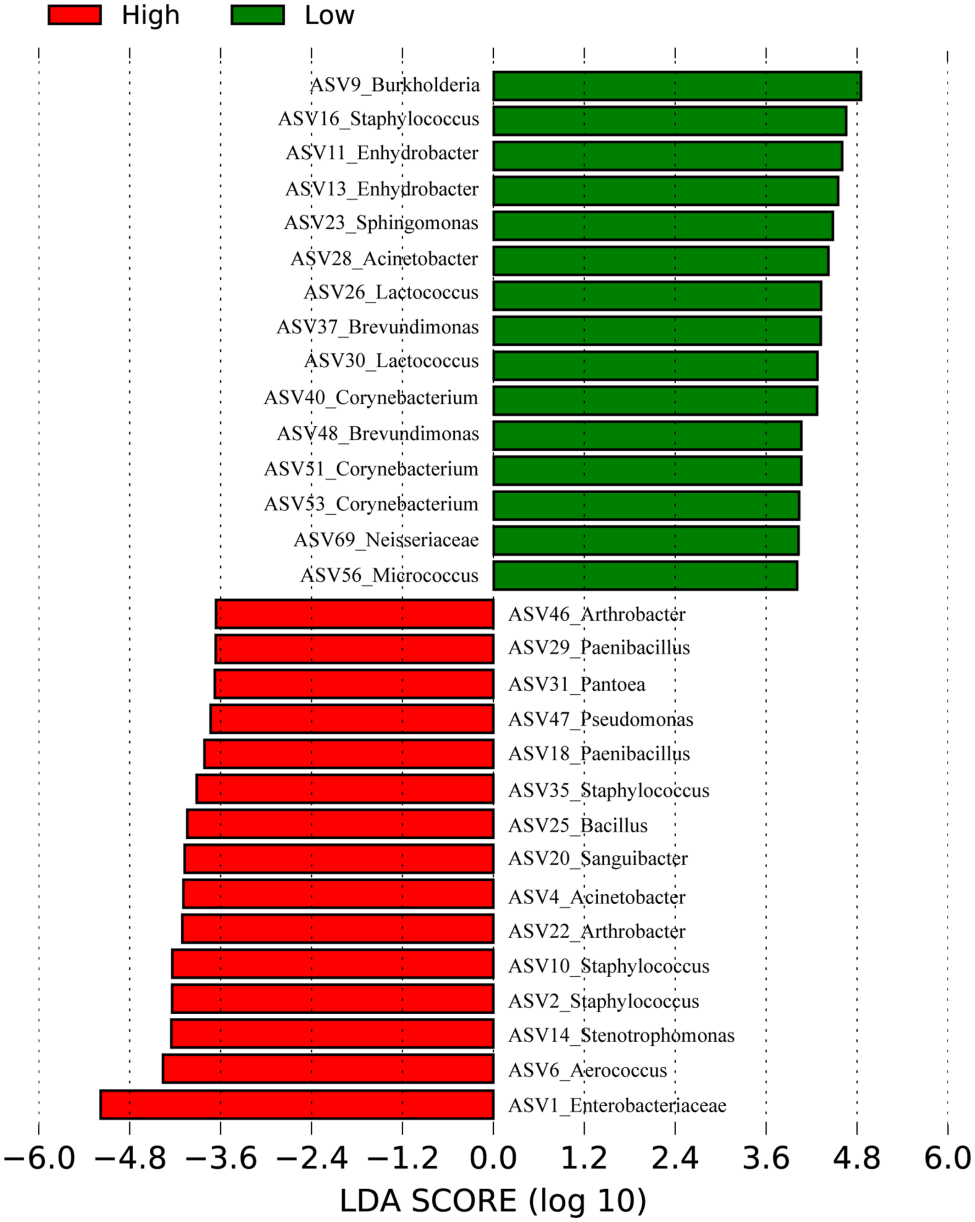
Bacteria differentiating high and low altitudes Humans by Linear discriminant analysis (LDA) coupled with effect size (LEfSe). Red represents the high-altitude group and green represents the low-altitude group.

Our previously published 16S rRNA data (Zeng et al., 2017) (128 samples) of skin samples from high and low- altitude humans were re-analyzed at the ASV level to identify signature bacteria between groups using linear discriminant analysis (LDA) effect size (LEfSe) (Figure 2). The main skin-featured bacteria at high altitude were *Aerococcus* (ASV6)*, Staphylococcus* (ASV2, ASV10, ASV35)*, Stenotrophomonas* (ASV14)*, Acinetobacter* (ASV4)*, Arthrobacter* (ASV22, ASV46)*, Sanguibacter* (ASV20)*, Pantoea* (ASV31)*, Bacillus* (ASV25)*, Paenibacillus* (ASV18, ASV29)*, Pseudomonas* (ASV47) and *Enterobacteriaceae unclassified* (ASV1). In the low altitude group, the main signature included *Burkholderia* (ASV9)*, Staphylococcaceae* (ASV16)*, Enhydrobacter* (ASV11, ASV13)*, Sphingomonas* (ASV23)*, Acinetobacter* (ASV28)*, Lactococcus* (ASV26, ASV30)*, Brevundimonas* (ASV37, ASV48)*, Corynebacterium* (ASV40, ASV51, ASV53)*, Micrococcus* (ASV56) and *Neisseriaceae unclassified* (ASV69).

### Culturability of high-altitude human skin microbes

**Figure 3:**
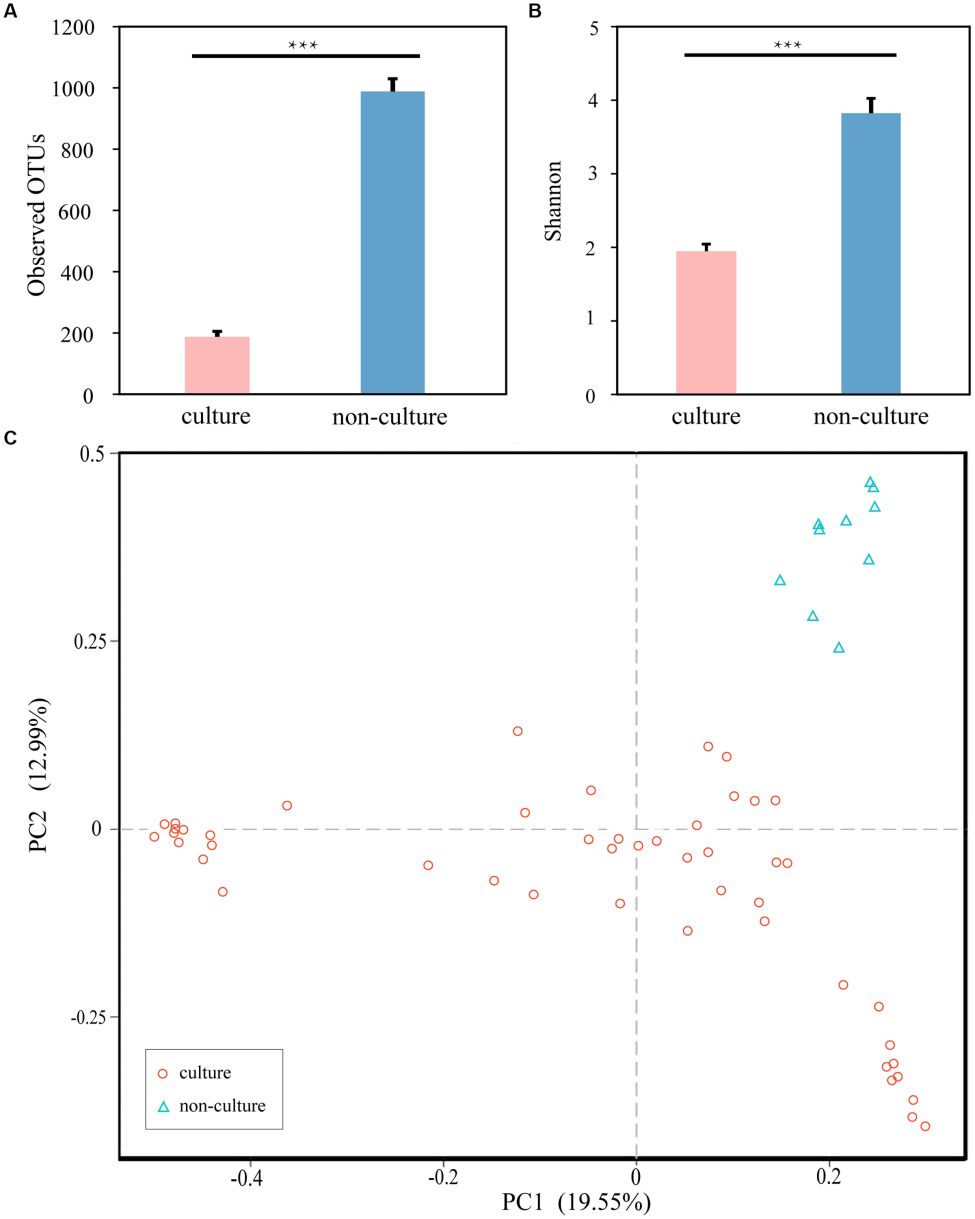
Microbial differences between non-culture and pure culture groups. **(A)** Observed OTUs. **(B)** Shannon Index. **(C)** PCoA plot based Bray-Curtis distance. Colors of red and blue represent samples on pure culture group and non-culture group. Pure culture group: using cultivation methods; non-culture group: using next-generation sequencing methods (***p < 0.05).

Next, to achieve the goal of bacterial isolation from high-altitude human skin that may resist and repair UV damage, the cultivation method using five different medias were performed. Compared to 1,438 ASVs from next-generation sequencing method (non-culture group), 1,054 ASVs were observed in the cultivation methods (pure culture group). In the meantime, 961 shared ASVs between the cultivation and next-generation sequencing methods (Supplementary Figure S1A), and then the culturability of high-altitude human skin microbes is 66.83% (961/1,438) at the ASV level. After annotation, we obtained 628 and 502 genera in the non-culture and pure culture groups, respectively, with 475 genera co-existing in both groups (Supplementary Figure S1B). Furthermore, non-culture group had higher alpha diversity than pure culture group (Figure 3). Correspondingly, beta diversity based on Bray-Curtis distance showed significant differences between pure culture (cult) and non-culture (skin).

**Figure 4:**
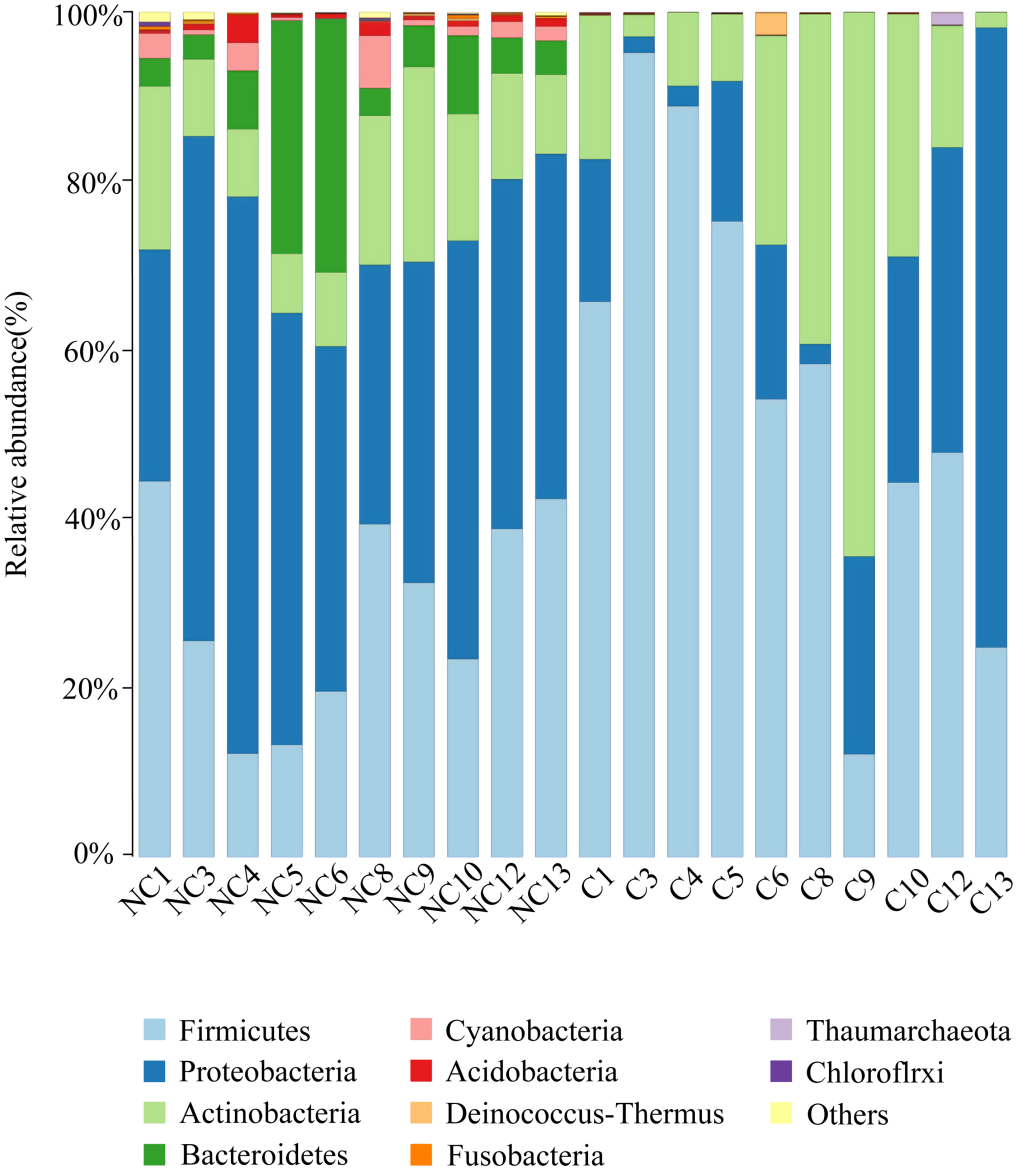
Microbial composition at the phylum level of each sample (top 10 taxon). NC (non-culture group): using next-generation sequencing methods; C (pure culture group): using cultivation methods. The same number represents from the same sample.

**Figure 5:**
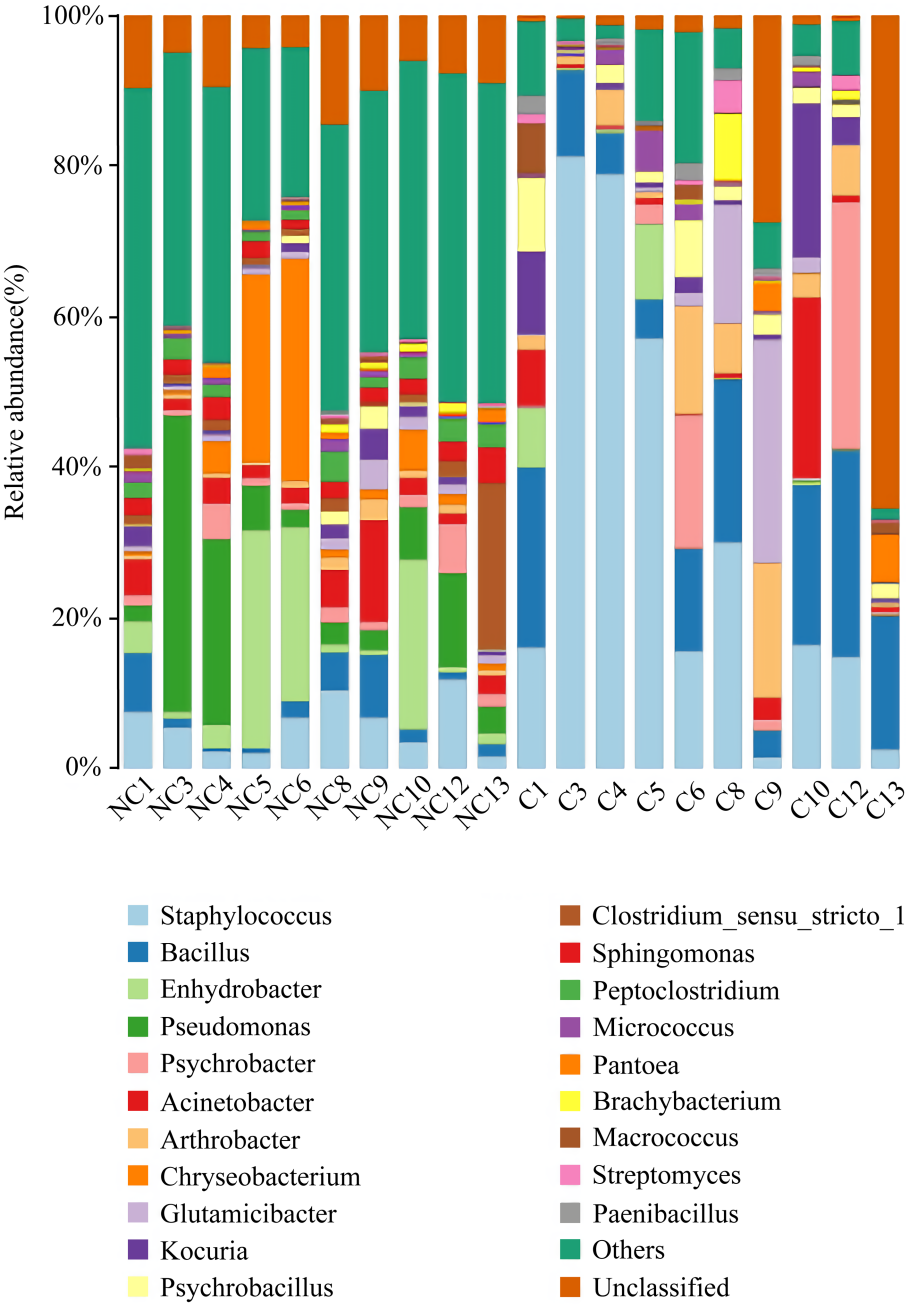
Microbial composition at the genus level of each sample (top 20 taxon). NC (non-culture group): using next-generation sequencing methods; C (pure culture group): using cultivation methods. The same number represents from the same sample.

At the phylum level, the dominant bacteria in the non-culture group were *Proteobacteria* (44.48%), *Firmicutes* (29.19%), *Actinobacteria* (13.04%), *Bacteroidetes* (9.57%) and *Acidobacteria* (0.92%) (Figure 4). The dominant genera were *Pseudomonas* (10.20%), *Enhydrobacter* (8.76%), *Chryseobacterium* (6.90%), *Staphylococcus* (5.85%), *Acinetobacter* (3.68%), *Clostridium sensu stricto 1* (3.32%), *Bacillus* (2.89%), *Sphingomonas* (2.37%), *Peptoclostridium* (2.35%), *Psychrobacter* (2.26%), *Glutamicibacter* (1.42%), *Kocuria* (1.31%) and *Arthrobacter* (1.12%) from the non-culture group (Figure 5). In the pure culture group, the dominant phyla were *Firmicutes* (55.82%), *Proteobacteria* (22.14%) and *Actinobacteria* (21.02%). Then, we identified 10 genera with relative abundance greater than 1%, which were considered to be the main genera that could be cultivated from all culture samples. These genera were *Staphylococcus* (30.81%), *Bacillus* (15.19%), *Arthrobacter* (5.65%), *Psychrobacter* (5.51%), *Glutamicibacter* (5.27%), *Kocuria* (4.09%), *Acinetobacter* (3.88%), *Psychrobacillus* (3.19%), *Enhydrobacter* (2.08%) and *Micrococcus* (1.27%).

There was a total of 72 genera with a relative abundance greater than 0.1% in the non-cultivation group, and except for *Endobacter*, we were able to acquire 71 genera through cultivation. Among these, 29 genera (Supplementary Table S3) of strains were obtained through isolated and purified culture. Additionally, we also obtained strains of 9 genera through isolated and purified culture, whose relative abundance was less than 0.1% in the non-culture group.

### Isolation of skin bacterial candidates to repair UV damage

We subsequently cultivated and isolated 340 single strains from these medias. Through sequencing the full-length of the 16S genes, a taxonomic map was generated. All isolations of strains were preliminarily divided into 4 phyla, 25 families, 42 genera, and 118 species (Table 1). We were able to cultivate 3 phyla (Firmicutes, Proteobacteria, Actinobacteria; *n* = 339), with Firmicutes (*n* = 175) and Actinobacteria (*n* = 148) being the most dominant (Figure 5). At the genus level, the majority of the isolated strains were *Kocuria* (*n* = 64), *Staphylococcus* (*n* = 54), *Bacillus* (*n* = 39), *Micrococcus* (*n* = 28), *Macrococcus* (*n* = 19), *Pantoea* (*n* = 16), *Rhodococcus* (*n* = 12), and *Paenibacillus* (*n* = 12). Furthermore, combining with results of Figure 2 and references, several bacterial candidates that may repair UV damage were selected for the below animal trial (Supplementary Table S3).

**Table 1:** Single bacteria obtained by isolation and purification.

Phylum	Family	Genus
*Actinobacteria*	*Brevibacteriaceae*	*Brevibacterium*
	*Micrococcaceae*	*Rothia*
		*Micrococcus*
		*Kocuria*
		*Arthrobacter*
	*Corynebacteriaceae*	*Carnobacterium*
		*Corynebacterium*
	*Microbacteriaceae*	*Curtobacterium*
		*Microbacterium*
	*Intrasporangiaceae*	*Janibacter*
		*Kytococcus*
	*Cellulomonadaceae*	*Oerskovia*
	*Dietziaceae*	*Dietzia*
	*Nocardioidaceae*	*Aeromicrobium*
	*Nocardiaceae*	*Rhodococcus*
	*Streptomycetaceae*	*Streptomyces*
*Proteobacteria*	*Moraxellaceae*	*Moraxella*
		*Acinetobacter*
		*Psychrobacter*
	*Enterobacteriaceae*	*Erwinia*
	*Methylobacteriaceae*	*Microvirga*
	*Rhodobacteraceae*	*Paracoccus*
		*Plantibacter*
	*Xanthomonadaceae*	*Stenotrophomonas*
*Firmicutes*	*Bacillaceae*	*Virgibacillus*
		*Oceanobacillus*
		*Solibacillus*
		*Psychrobacillus*
		*Lysinibacillus*
		*Exiguobacterium*
		*Bacillus*
	*Carnobacteriaceae*	*Desemzia*
	*Enterococcaceae*	*Enterobacter*
	*Enterobacteriaceae*	*Pantoea*
	*Staphylococcaceae*	*Staphylococcus*
		*Macrococcus*
	*Paenibacillaceae*	*Brevibacillus*
		*Paenibacillus*
	*Planococcaceae*	*Planomicrobium*
	*Planococcaceae*	*Sporosarcina*
	*Aerococcaceae*	*Aerococcus*
*Deinococcus Thermus*	*Deinococcaceae*	*Deinococcus*

### Animal trial to test UV-damage repair isolations

To test UV damage repair functions of isolations, bacterial suspension of *Arthrobacter gandavensis, Bacillus psychrosaccharolyticus, Pantoea eucrina, Paenibacillus amylolyticus* and *Paenibacillus terrae* were applied to the mice skin hurt by UV lamp. The skin of all the negative control mice visually thickened, and numerous inflammatory cells infiltrated in the dermis and subcutaneous tissue layers. The skin pathological changes in experimental groups (A, B, D, E) were similar to those in the negative control group (Supplementary Figure S2). We scored the skin pathological changes of each mouse in the groups (Figures 1A–D) and found that experimental group C (*Pantoea eucrina*) had the lowest pathological score except blank control group, indicating the lowest degree of skin damage (for detailed score statistics, Supplementary Table S7) (Figure 1E). Notably, there was a significant difference (*p* = 0.027) in the skin pathological section scores between the experimental group C and the negative control group, with only a small amount of inflammatory cell infiltration in the dermis and subcutaneous tissue layers (Figure 1B). Therefore, the bacteria in group C (*Pantoea eucrina*) have the potential to repair skin UV damage.

### Validation of UV-resistant functions of *Pantoea eucrina KBFS172*

**Figure 6:**
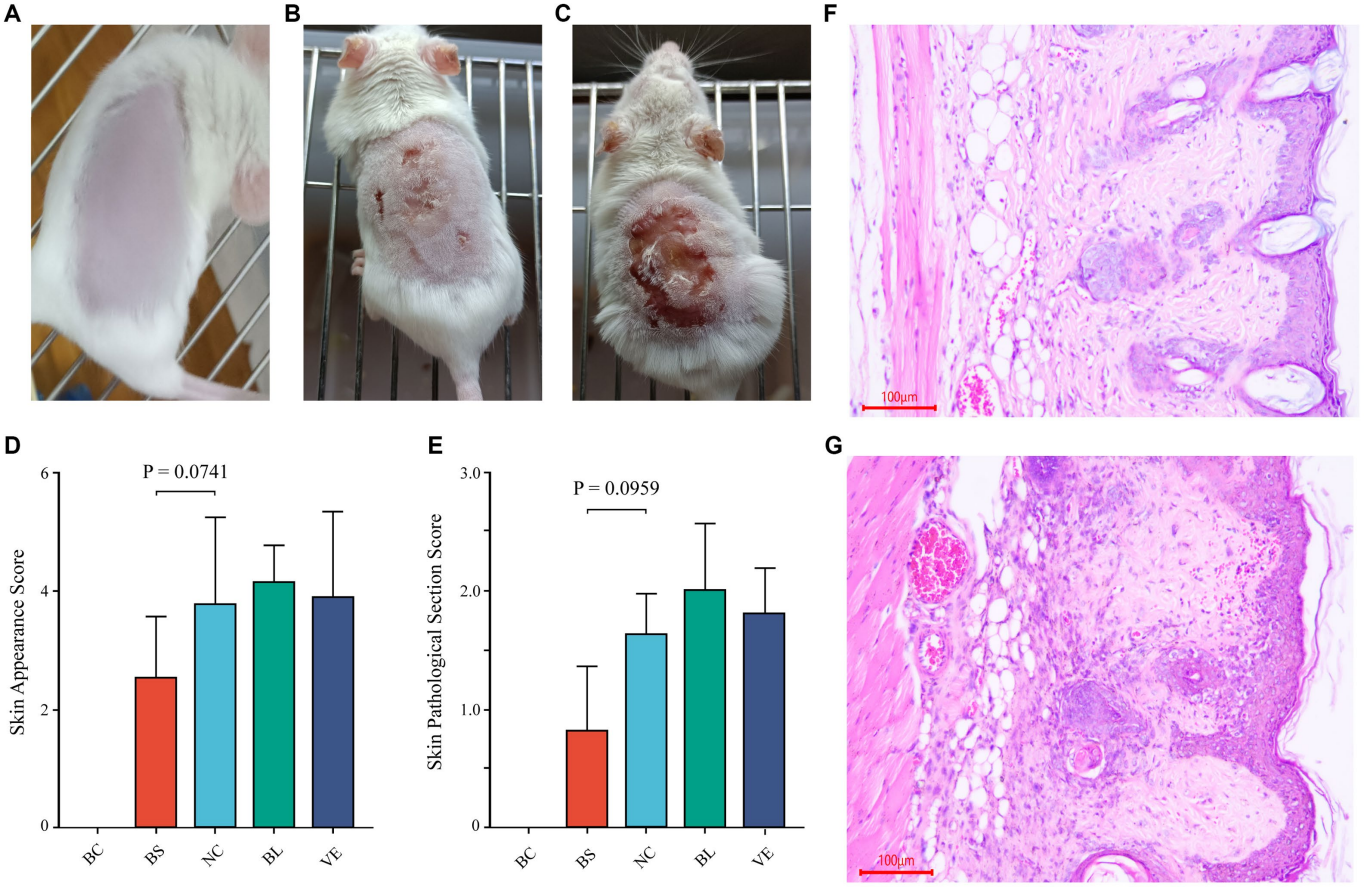
Mice trial #2: pathological changes of skin and sections, and scoring of skin pathological changes. **(A)** The healthy mouse skin appearance (BC). **(B)** Appearance of the skin of the mice smeared with BS. **(C)** Appearance of the skin of the mice in NC. **(D, E)** Statistical bar graph of skin appearance and skin pathological damage score in each group. **(F)** Representative diagram of skin section in the BS. **(G)** Representative diagram of skin section in the NC. BC, blank control; BS, bacterial suspension: Suspension of *Pantoea eucrina*; NC, negative control; BL, bacterial lysate: lysate of *Pantoea eucrina*; VE, vitamin E.

To further investigate whether the *Pantoea eucrina* strain or its metabolites could repair skin UV damage, a mice trial #2 was conducted. Firstly, in visual observation, the skin of the mice in the BS group (bacterial suspension: Suspension of *Pantoea eucrina*) appeared chapped erythema, while other control groups (NC group: negative control, BL group: bacterial lysate: lysate of *Pantoea eucrina*, and VE group: vitamin E, Supplementary Table S4) showed varying degrees of ulceration, dandruff production, and scarring. From the appearance of the mice skin, it appeared that the skin damage situation of the mice in the BC group was indeed better than that in the NC group (Figures 6B,C). The appearance and tissue pathological damage scores in BS groups were the best compared to NC Group, BL Group, and VE group (Figure 6), and the trial details were documented in Supplementary Tables S4–S6, S8, S9, and Supplementary Figure S2. In addition, we observed that BS groups had a better UV-resistant effect than NC and BL groups, indicating that *Pantoea eucrina* strain plays critical roles in UV-resistant rather than its metabolites.

### Functional annotation of *Pantoea eucrina KBFS172* using whole genome sequencing

**Figure 7:**
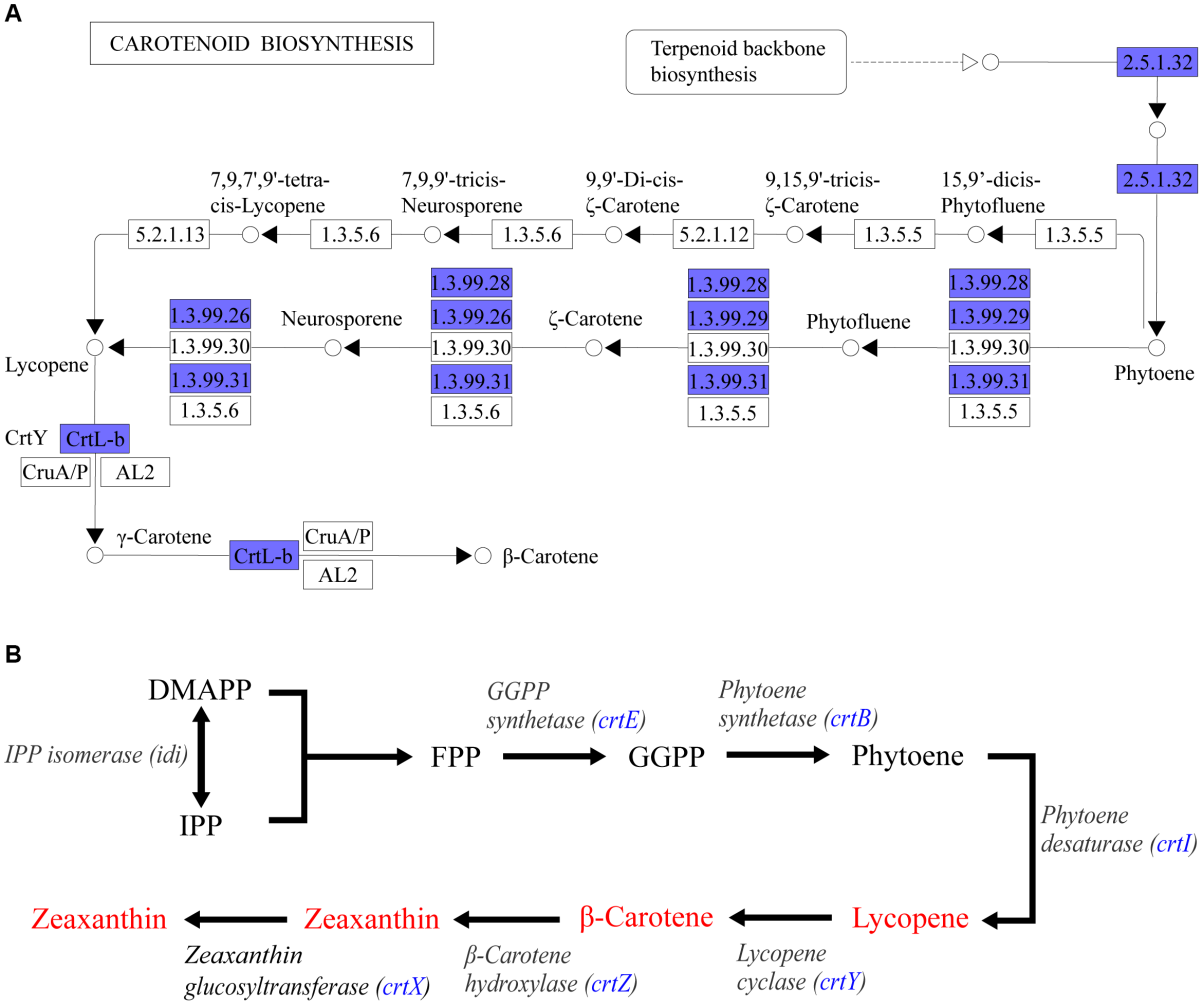
KEGG pathway enrichment analysis annotation results. **(A)** Beta-Carotene biosynthesis module (M00097) in carotenoid biosynthesis metabolic pathway. Blue filled were the annotated genes in *Pantoea eucrina KBFS172*. **(B)** Sketch of carotenoid synthesis pathway inferred from *P. agglomerans KFS-9* and *Pantoea ananatis*. The blue filled was the enzyme annotated by *Pantoea eucrina KBFS172*.

Through whole genome sequencing technology, a total of 4,053,112 bases of *Pantoea eucrina KBFS172* was generated, which included 1 chromosome sequence and 4 plasmid sequences, and predicted 3,871 genes (Supplementary Table S10). A total of 107 potential functional pathways were recognized and quantified, which included the metabolic pathways of carotenoid synthesis, ascorbate metabolism, geraniol degradation and streptomycin biosynthesis, etc. Carotenoids have been shown to act as an antioxidant against oxidative stress and increase bacterial tolerance to UV radiation (Ordenes-Aenishanslins et al., 2016; Portero et al., 2019; Reis-Mansur et al., 2019; Sedlacek et al., 2019). In this study, *Pantoea eucrina KBFS172* had a large number of genes enriched in the metabolic pathway of carotenoid biosynthesis (Supplementary Figure S4, S5), among which beta-Carotene biosynthesis module was particularly prominent (Figure 7A). In addition, 6 enzymes were annotated to be closely related to carotenoid biosynthesis, including *geranylgeranyl pyrophosphate synthase (crt_E; GE03214), zeaxanthin glucosyltransferase (crt_X; GE03215), lycopene cyclase (crt_Y; GE03216), phytoene dehydrogenase (crt_I; GE03217), phytoene synthase (crt_B; GE03218), and beta-carotene hydroxylase (crt_Z; GE03219)*. Through consultation of the key gene set for carotenoid synthesis in *Pantoea* (Wang et al., 2007; Mohammadi et al., 2012; Choi et al., 2021), we speculated that these 6 enzymes were involved in the pathway of carotenoid biosynthesis in the order shown in Figure 7B.

## Discussion

Although many studies focus on isolations of beneficial bacteria of gut microbiota (Deng et al., 2023; Xu et al., 2023; Zhao et al., 2023), the crucial role of skin microbes in humans have been highlighted. Interactions between certain skin microbiome and host cells can result in functional changes in the latter. Past research has shown that skin microbes are involved in anti-inflammatory processes, maintenance of skin homeostasis, and regulation of the immune system in both humans and animals (Ridaura et al., 2018).

Four dominant bacterial phyla in the human skin microbiota had been identified, including *Proteobacteria, Actinobacteria, Firmicutes, and Bacteroidetes* (Grice et al., 2008; Li et al., 2019). Changes in the living environment could affect the skin microbial composition. A previous study found that the relative abundance of *Bacteroidetes* and *Firmicutes* was higher at high altitudes (Li et al., 2019) than at low altitudes (Grice et al., 2008). This difference suggests that altitude may be an important driving force for skin microbiota composition (Gao et al., 2007; Fierer et al., 2008; Grice et al., 2009). Our own results showed that the relative abundance of continued to increase compared to the previous study. Therefore, *Firmicutes* might be beneficial for humans living at high altitudes. At the genus level, previous studies have found the resident microbiota in human skin, such as *Propionibacterium, Corynebacterium, Staphylococcus, Streptococcus, Acinetobacter, Colwiebacteria,* and *Enhydrobacter* (Gao et al., 2007; Fierer et al., 2008; Grice et al., 2009; Leung et al., 2015). *Propionibacterium* and *Staphylococcus* have been found to be the predominant species in sebaceous sites (Grice et al., 2008). However, in our study, *Propionibacterium* was not found to be predominant and its relative abundance was less than 1%, suggesting that it may not be able to tolerate the high-altitude environment. Interestingly, we found two genera, *Pseudomonas* (10.20%, *Proteobacteria*) and *Chryseobacterium* (6.90%, *Bacteroidetes*), with high relative abundance that had rarely been found at such levels in previous studies on human skin. This indicates that these two genera may be tolerant to high-altitude environments. Previous studies (Seelam et al., 2021) have demonstrated that *Pseudomonas* has the potential ability to provide protective effects mediated by melanin as a sunscreen agent against UV-B radiation. However, *Pseudomonas* has also been reported as an opportunistic pathogen of human skin (Spernovasilis et al., 2021), *Chryseobacterium* has been less studied on human skin, but a few studies (Gurav and Jadhav, 2013; Venil et al., 2015; Hui et al., 2019) have reported that its metabolite flexirubins can treat chronic skin diseases, while other studies (Nulens et al., 2001; Cascio et al., 2005) have shown that it is closely associated with some human diseases. Therefore, further research is needed to elucidate the specific functions of these two genera at the species level.

In our study, we employed the cultivation method using five different media and were able to obtain approximately 66.83% (961/1,438) of operational taxonomic units (OTUs). However, in the non-cultured group, we observed that five genera, including *Pseudomonas, Chryseobacterium, Clostridium sensu stricto 1, Sphingomonas*, and *Peptoclostridium*, had a relative abundance greater than 1% but were uncultured. These genera need to be further cultivated using other media, particularly *Pseudomonas* (isolated referencing (Ravi et al., 2018)) and *Chryseobacterium* (isolated referencing (Nishioka et al., 2016)), which are speculated from our results to be closely associated with high altitude. The skin bacteria that we were able to cultivate were dominated by *Firmicutes*, with most isolates belonging to *Staphylococcus* (30.81%) followed by *Bacillus* (15.19%). These bacteria were relatively common (Timm et al., 2020; Fleming et al., 2021). Certainly, culturability is closely related to the culture conditions. Thus, more culture media and different culture conditions can be employed to further cultivate uncultivated bacteria.

Until now, there have been relatively few studies conducted on the skin microbiota of Tibetans living on the plateau. Our study has provided insight into the structure of the skin microbiota of Tibetans living on the plateau, with a focus on the culturomics of extremely rare high-altitude human skin microbes. This study serves as a reference for future investigations into human skin microbes. Additionally, we observed that approximately 66% of plateau Tibetan skin microbes could be cultivated using five different media (Supplementary Table S2). Although some of the isolated strains may be more commonly associated with soil or environmental sources, they were nonetheless found on the skin of healthy individuals. The contribution of these strains to the function of the skin microbiome is unknown, and further research in this area is warranted.

To further explore the function of skin microbes in plateau Tibetans and identify strains that can repair UV damage, we conducted mice trials using five selected strains from pure culture based on previous studies. Ultimately, we identified a strain (*Pantoea eucrina KBFS172*) that showed ability in repairing photodamage. Previous studies have demonstrated the photodamage repair function of skin bacteria, and they have multiple repair mechanisms, which have different repair mechanisms. Some studies have found that the metabolites of certain microorganisms can achieve the function of photodamage repair or alleviation, such as bioactive peptides (Wang et al., 2022), Vitamin C, ferulic acid, and phloretin, etc. (Oresajo et al., 2008). In our study, *Pantoea eucrina* was identified to associate with the metabolism of carotenoids. Carotenoids have been shown to have anti-oxidative stress properties, increase bacterial UV radiation tolerance (Ordenes-Aenishanslins et al., 2016; Reis-Mansur et al., 2019), and found that co-cultivation of carotenoids with bacteria can enhance the UV radiation tolerance of sensitive bacteria (Portero et al., 2019; Sedlacek et al., 2019). Additionally, we also found other species of the genus *Pantoea*, which could also produce carotenoids, such as *Pantoea ananatis, Pantoea stewartii subsp. Stewartii, and P. agglomerans Eho10* (Mohammadi et al., 2012; Sedlacek et al., 2019; Choi et al., 2021). This indicated that the *Pantoea* genus may have excellent potential for repairing photodamage. Moreover, *Micrococcus* (Hofer et al., 2011), *cyanobacteria* (Stege, 2001), and *Lactobacillus acidophilus* (Im et al., 2018) can also repair skin UV damage by synthesizing DNA damage repair enzymes or photolytic enzymes. Therefore, skin microbes can reduce the damage of ultraviolet rays to human skin in a variety of ways.

## Conclusion

In conclusion, we characterized the skin microbiota of humans living in high altitude regions of China using 16S rRNA gene sequencing and culturomics methods. We identified and isolated strains that were effective in repairing UV damage through mouse trials, and obtained a potentially functional strain. The function of the strain was then verified by analyzing the whole-genome sequencing results. These findings gave the insights for further studies to explore the potential of skin-associated microbial communities in high altitude adaptation of humans, and offer new insights into the development of human skin probiotic products to resist skin diseases, such as skin cancer or sunburn.

## Data availability statement

The rumen metagenome sequences were deposited into NCBI Sequence Read Archive (SRA) under the accession number PRJNA1002829. The other sequences data in the study are included in the article/Supplementary material, and further inquiries can be directed to the corresponding authors.

## Ethics statement

The experiment protocol was approved by the Animal Ethics and Humane Animal Care of the Foshan University. The studies were conducted in accordance with the local legislation and institutional requirements. Written informed consent for participation in this study was provided by the participants’ legal guardians/next of kin. Written informed consent was obtained from the owners for the participation of their animals in this study.

## Author contributions

ZZ: Data curation, Formal analysis, Investigation, Methodology, Writing – original draft. HR: Investigation, Writing – original draft. YH: Investigation, Writing – review & editing. FD: Resources, Visualization, Writing – review & editing. BZ: Investigation, Resources, Validation, Visualization, Writing – original draft. JC: Supervision, Validation, Visualization, Writing – review & editing. YL: Conceptualization, Funding acquisition, Project administration, Supervision, Validation, Visualization, Writing – original draft, Writing – review & editing.
