# Supplementary material for: Screening and evaluation of skin potential probiotic from high-altitude Tibetans to repair ultraviolet radiation damage
Source: Front Microbiol. 2023 Oct 19;14:1273902. doi: 10.3389/fmicb.2023.1273902 (PMC10620709; doi:10.3389/fmicb.2023.1273902)
Supplement: Supplementary file 1 [file Data_Sheet_1.docx]

**Supplementary Table S1.** The characteristics of samples

| Subject ID | Gender | Age | Sampling site | Latitude & Longitude |
| --- | --- | --- | --- | --- |
| M3B | female | middle | forehead | latitude 28°75' N, longitude 100°17' E |
| M11A | male | middle | forehead | latitude 28°75' N, longitude 100°17' E |
| M49 | male | middle | forehead | latitude 28°75' N, longitude 100°17' E |
| M24 | male | middle | forehead | latitude 28°75' N, longitude 100°17' E |
| M14A | male | child | forehead | latitude 28°75' N, longitude 100°17' E |
| M7A | female | elderly | forehead | latitude 28°75' N, longitude 100°17' E |
| M10A | male | elderly | forehead | latitude 28°75' N, longitude 100°17' E |
| M5A | female | elderly | forehead | latitude 28°75' N, longitude 100°17' E |
| M70 | female | elderly | forehead | latitude 28°75' N, longitude 100°17' E |
| M9A | female | middle | forehead | latitude 28°75' N, longitude 100°17' E |

Note:

Middle 30-50

Elderly:>=60

**Supplementary Table S2.** List of media

| Media | Reagent (g/L) | Reference |
| --- | --- | --- |
| Nutrient Agar | Peptone, 10g; Beef Extract, 3g; Sodium chloride, 5g; Agar, 15g | (Van der Weele et al. 2000) |
| R2A Agar | Yeast extract, 0.5g; Peptone, 0.5g; Pancreatic digest of casein, 0.5g; Starch, 0.5g; Dextrose, 0.5g; Dipotassium phosphate, 0.3g; Sodium pyruvate, 0.3g; Magnesium sulfate, 0.024g; Agar, 15g | (Osawa and Microbiology 1990) |
| TSBYS | Yeast extract, 3g; Tryptone, 17g; Peptone, 3g; Dextrose, 2.5g; Sodium chloride, 18g; Dipotassium phosphate, 2.5g; Potassium chloride, 0.34g; Magnesium chloride hexahydrate, 4g; Magnesium sulfate heptahydrate, 3.45g; Ammonium chloride, 0.25g; Calcium chloride dihydrate, 0.14g | (Nicholson et al. 2013, Peeters et al. 2011) |
| Brain-Heart Infusion Agar (BHIA) | Calf brains Infusion from 200g, 4g; Beef heart Infusion from 250g, 4g; Peptone, 5g; Proteose peptone, 16g; Sodium chloride, 5g; Dextrose, 2g; Disodium phosphate, 2.5g; Agar, 15g | (Osawa and Microbiology 1990) |
| Agar medium J (Deoxycholate Citrate Agar) | Saccharose, 50g; Ammonium nitrate, 2.5g; Potassium dihydrogen phosphate, 1.5g; Calcium chloride, 0.1g; Magnesium sulfate, 1g; Yeast extract, 3g | (Pagnanelli et al. 2000) |

**Supplementary Table** **S3.** Group information and processing conditions in mice trial 1.

| Groups | UV | Colonized species | Dose | Number of mice |
| --- | --- | --- | --- | --- |
| Negative | F | \ | \ | 3 |
| Positive | T | Nutrient broth | \ | 5 |
| Group A | T | *Arthrobacter gandavensis* | 1.0×10^8^CFU/mL | 5 |
| Group B | T | *Bacillus psychrosaccharolyticus* | 1.0×10^8^CFU/mL | 5 |
| Group C | T | *Pantoea eucrina* | 1.0×10^8^CFU/mL | 5 |
| Group D | T | *Paenibacillus amylolyticus* | 1.0×10^8^ CFU/mL | 5 |
| Group E | T | *Paenibacillus terrae* | 1.0×10^8^ CFU/mL | 5 |

**Supplementary Table S4.** Group information and processing conditions in mice trial 2.

| Group | Number | Ultraviolet irradiation | | Treatment |  |
| --- | --- | --- | --- | --- | --- |
| BC group | 1-8 | F | | NA |  |
| NC group | 9-16 | T | | 1 ml of sterile culture medium |  |
| BS group | 17-24 | T | Smear 1ml of the bacterial suspension of *Pantoea eucrina KBFS172* | | |
| BL Group | 25-32 | T | | Smear 1 ml of the lysate |  |
| VE group | 33-40 | T | | Smear vitamin E |  |
| BC (blank control); BS (bacterial suspension: Suspension of *Pantoea eucrina*); NC (negative control); BL (bacterial lysate: lysate of *Pantoea eucrina*); VE (vitamin E) | | | | |  |

**Supplementary Table S5.** UV damage in mice dorsal skin condition score.

| Score | Skin status |
| --- | --- |
| 0 | smooth, plump and elastic |
| 1 | Wrinkles obviously; redness on skin |
| 2 | Deep wrinkles; small amount of chap |
| 3 | Chap and erythema obviously |
| 4 | Scars obviously; a lot of erythema and dander; laxity |
| 5 | Skin thickening and ulceration |

Corresponding to Figure 4 (F.a-f) in turn

**Supplementary Table S6.** Histopathological damage scoring standard.

| Grade | Degree of damage | Inflammatory cells infiltration | Score |
| --- | --- | --- | --- |
| - | No visual difference between epidermal cell thickness and normal skin thickness. | No pathological damage | 0 |
| + | Epidermis is twice as thick as normal skin. | Sporadic inflammatory cell infiltration | 1 |
| ++ | Epidermis is three as thick as normal skin; obvious protrusions in dermis or subcutaneous tissue. | A small amount of inflammatory cells infiltration | 2 |
| +++ | Epidermis thicker than normal skin three times; a number of huge protrusions in dermis or subcutaneous tissue. | A large number of inflammatory cells infiltration | 3 |

Corresponding to Figure 4 (A-D) in turn

**Supplementary Table S7.** Mice skin tissue pathological damage score in trial 1.

| Group | Normal (0) | Mild (1) | Medium (2) | Severe (3) | Score |
| --- | --- | --- | --- | --- | --- |
| Negative CG | +++ |  |  |  | 0 |
| Blank C |  | + | + | +++ | 12 |
| Group A |  | ++ | +++ |  | 8 |
| Group B |  | + | +++ | + | 10 |
| Group C |  | ++++ | + |  | 6 |
| Group D |  | + | +++ | + | 10 |
| Group E |  | + | +++ | + | 10 |
| Group F |  | ++ | + | ++ | 10 |

**Supplementary Table S8.** Mice skin appearance damage score in trial 2

| Number  Group | 1 | 2 | 3 | 4 | 5 | 6 | 7 | 8 | Score |
| --- | --- | --- | --- | --- | --- | --- | --- | --- | --- |
| BC group | 0 | 0 | 0 | 0 | 0 | 0 | 0 | 0 | 0 |
| NC group | 3 | 5 | 1 | 3 | 5 | 5 | 5 | 3 | 30 |
| BS group | 4 | 2 | 2 | 1 | 4 | 2 | 3 | 2 | 20 |
| BL group | 4 | 4 | 4 | 4 | 3 | 5 | 5 | 4 | 33 |
| VE group | 4 | 5 | 3 | 5 | 3 | 5 | 5 | 1 | 31 |
| BC (blank control); BS (bacterial suspension: Suspension of *Pantoea eucrina*); NC (negative control); BL (bacterial lysate: lysate of *Pantoea eucrina*); VE (vitamin E) | | | | | | | | | |

**Supplementary Table S9.** Mice skin tissue pathological damage score in trial 2.

| Group | Normal (0) | Mild (1) | Moderate (2) | | Severe (3) | | Score |
| --- | --- | --- | --- | --- | --- | --- | --- |
| BC group | ++++++ |  |  |  | | 0 | |
| NC group |  | +++ | ++ | + | | 10 | |
| BS group | ++ | +++ | + |  | | 5 | |
| BL group |  | ++ | ++ | ++ | | 12 | |
| VE group |  | ++ | +++ | + | | 11 | |
| BC (blank control); BS (bacterial suspension: Suspension of *Pantoea eucrina*); NC (negative control); BL (bacterial lysate: lysate of *Pantoea eucrina*); VE (vitamin E) | | | | | | | |

**Supplementary Table S10. The genome assembly results statistics.**

| Sample | Scaffold Length (bp) | Scaffold Number | Scaffold Number | Scaffold N90 (bp) | Contig Length (bp) | Contig N50 (bp) | Contig N90 (bp) | GC content (%) |
| --- | --- | --- | --- | --- | --- | --- | --- | --- |
| Sample1 | 4,053,112 | 5 | 3,365,778 | 388,872 | 4,053,112 | 3,365,778 | 388,872 | 55.91 |


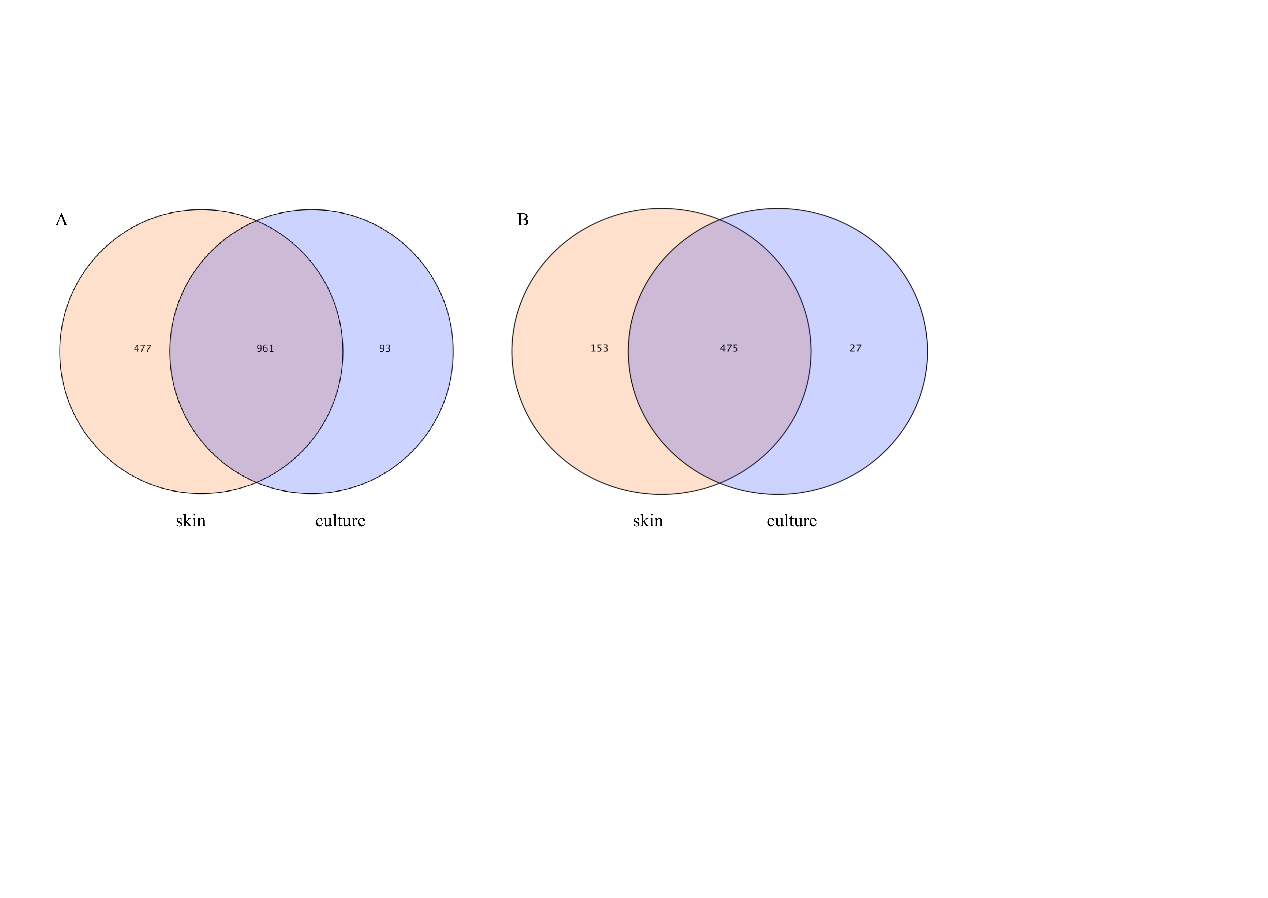


**Supplementary Figure S1.** Venn diagram. The intersection of microbiota between direct sequencing and culture at the OTU level (A) OTU level (B) genus level


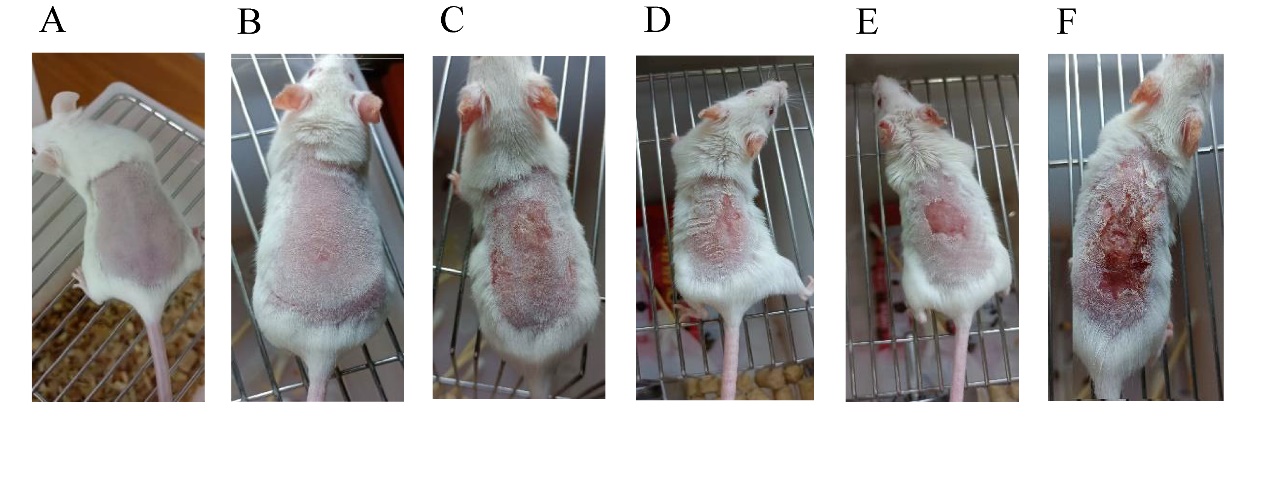


**Supplementary Figure S2.** The appearance of skin damage in mice, A-F corresponding to 0-5 points respectively.
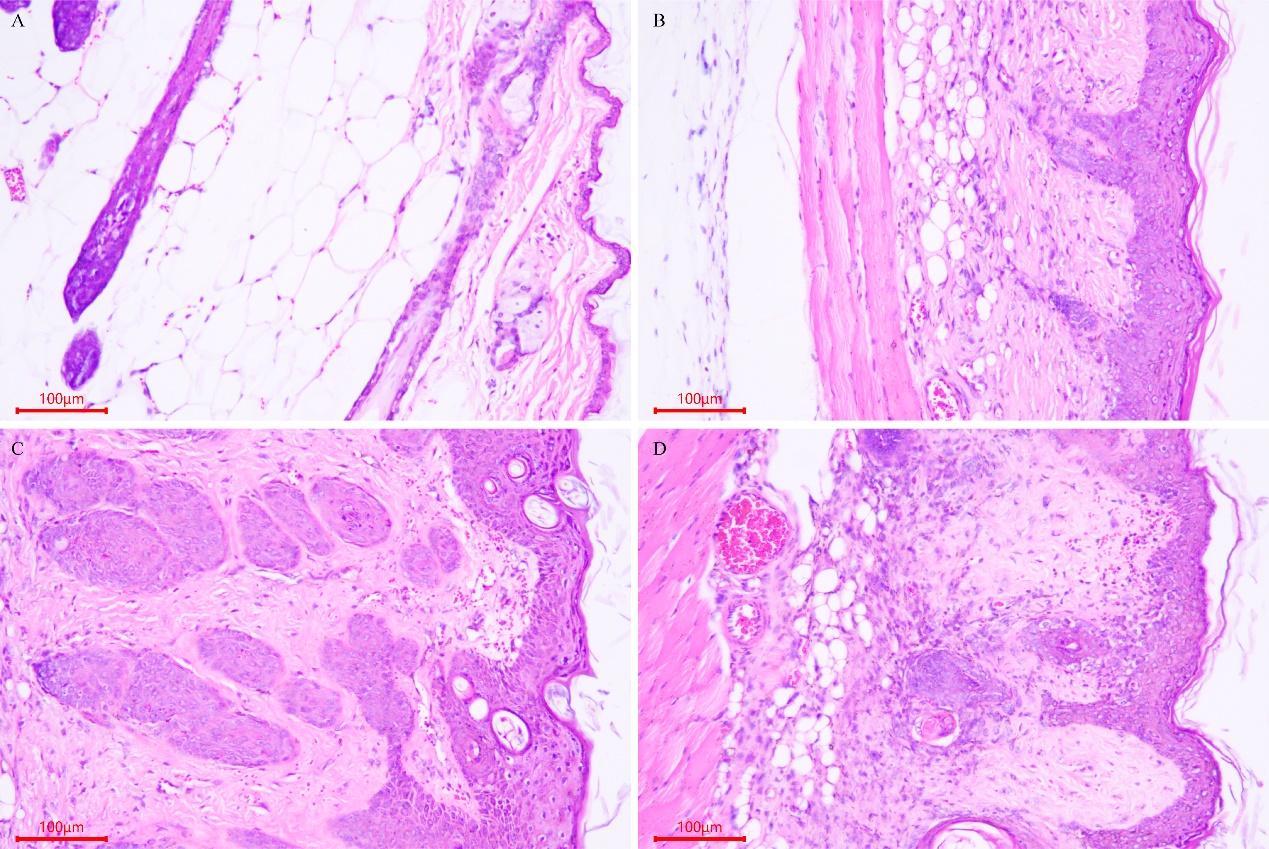


**Supplementary Figure S3.** Mouse skin pathological slices. A-D correspond to pathological damage scores 0-3 respectively.


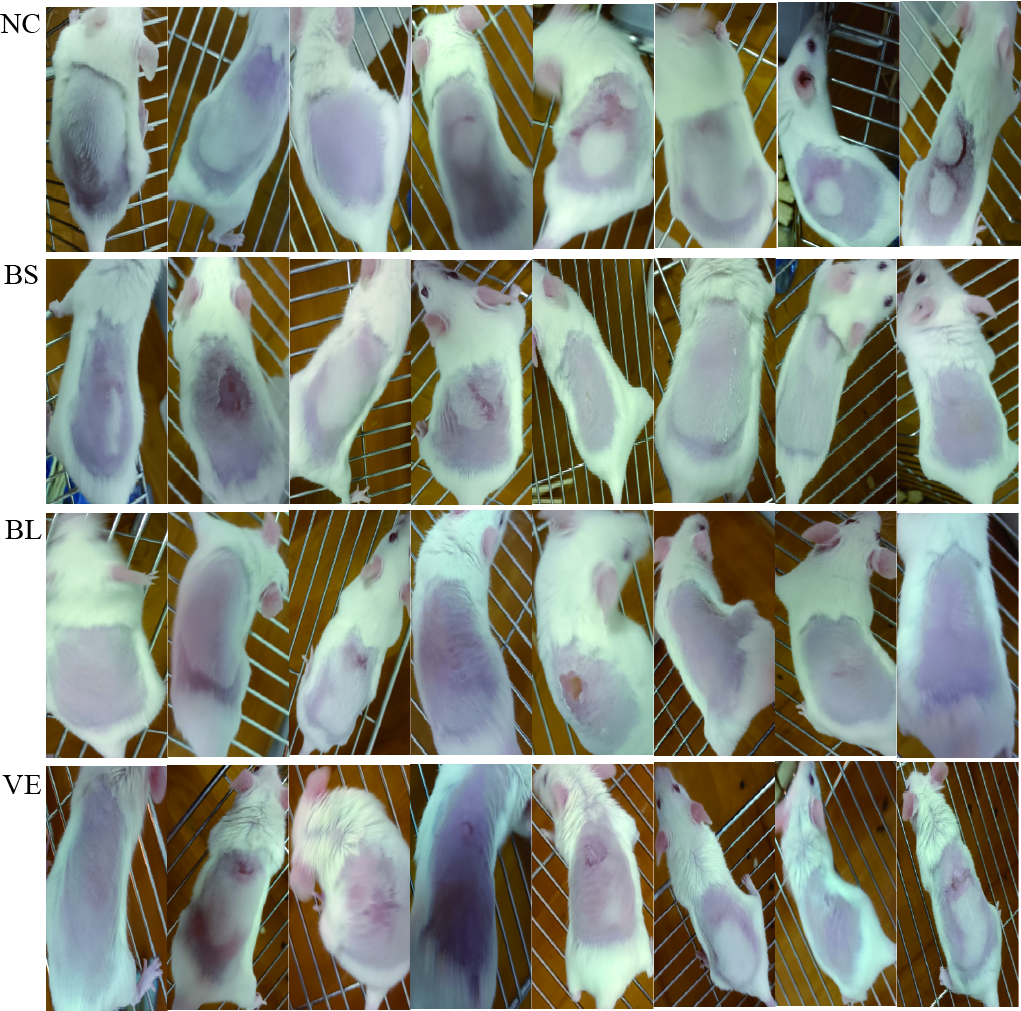


**Supplementary Figure S4.** The back skin condition of mice in each group during the first week following the completion of Mice UV Irradiation Trial #2


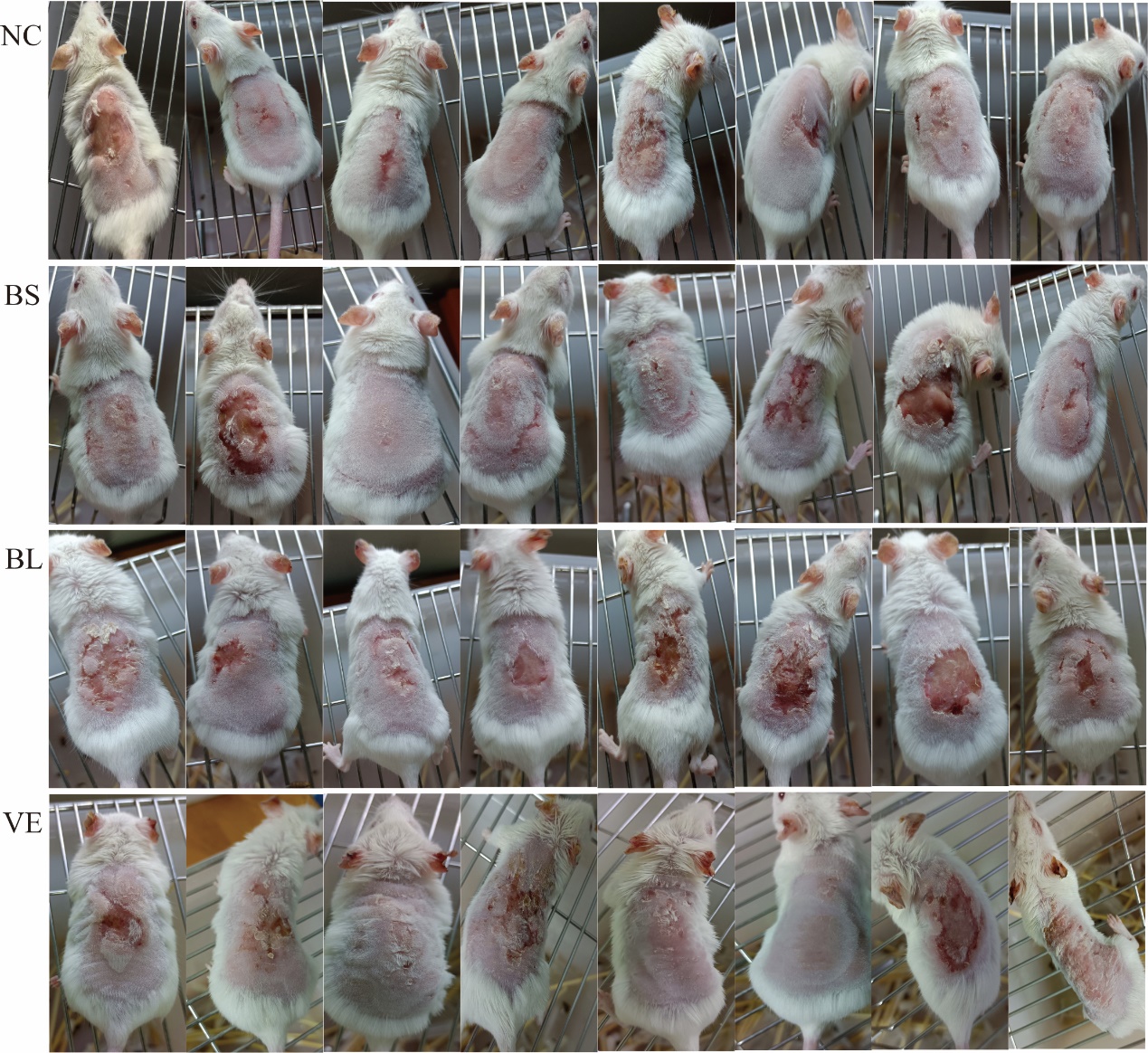


**Supplementary Figure S5.** The back skin condition of mice in each group during the third week following the completion of Mice UV Irradiation Trial #2

Reference

Nicholson, Wayne L, Kirill Krivushin, David Gilichinsky, and Andrew C %J Proceedings of the National Academy of Sciences Schuerger. 2013. "Growth of Carnobacterium spp. from permafrost under low pressure, temperature, and anoxic atmosphere has implications for Earth microbes on Mars." 110 (2):666-671.

Osawa, R %J Applied, and Environmental Microbiology. 1990. "Formation of a clear zone on tannin-treated brain heart infusion agar by a Streptococcus sp. isolated from feces of koalas." 56 (3):829-831.

Pagnanelli, F, M Petrangeli Papini, L1 Toro, M Trifoni, F %J Environmental Science Veglio, and Technology. 2000. "Biosorption of metal ions on Arthrobacter sp.: biomass characterization and biosorption modeling." 34 (13):2773-2778.

Peeters, Karolien, Dominic A Hodgson, Peter Convey, and Anne %J Microbial Ecology Willems. 2011. "Culturable diversity of heterotrophic bacteria in Forlidas Pond (Pensacola Mountains) and Lundström Lake (Shackleton Range), Antarctica." 62 (2):399-413.

Van der Weele, Corine M, William G Spollen, Robert E Sharp, and Tobias I %J Journal of experimental botany Baskin. 2000. "Growth of Arabidopsis thaliana seedlings under water deficit studied by control of water potential in nutrient‐agar media." 51 (350):1555-1562.
